# Recent Progress in Light-Driven Molecular Shuttles

**DOI:** 10.3389/fchem.2021.832735

**Published:** 2022-02-02

**Authors:** Bin Yao, Hongfei Sun, Lin Yang, Song Wang, Xingyan Liu

**Affiliations:** Chongqing Key Laboratory of Catalysis and New Environmental Materials, College of Environment and Resources, Chongqing Technology and Business University, Chongqing, China

**Keywords:** molecular shuttles, photoisomerization, photochemical reaction, photoinduced electron transfer, catalysis, drug delivery, ion transport

## Abstract

Molecular shuttles are typical molecular machines that could be applied in various fields. The motion modes of wheel components in rotaxanes could be strategically modulated by external stimuli, such as pH, ions, solvent, light, and so on. Light is particularly attractive because it is harmless and can be operated in a remote mode and usually no byproducts are formed. Over the past decade, many examples of light-driven molecular shuttles are emerging. Accordingly, this review summarizes the recent research progress of light-driven molecular shuttles. First, the light-driven mechanisms of molecular motions with different functional groups are discussed in detail, which show how to drive photoresponsive or non-photoresponsive molecular shuttles. Subsequently, the practical applications of molecular shuttles in different fields, such as optical information storage, catalysis for organic reactions, drug delivery, and so on, are demonstrated. Finally, the future development of light-driven molecular shuttle is briefly prospected.

## Introduction

Recently, mechanically interlocked molecules (MIMs) have received widespread attention for their well-defined and unique interlocked structures and appealing stimuli-responsive properties in various fields particularly in artificial molecular machines and nanoscience (Erbas-Cakmak et al., 2015; Stoddart 2017; Sun et al., 2019; Dattler et al., 2020; Cai et al., 2021). Rotaxane is one type of the most crucial representative of MIMs, which is often composed of an axle component interlocked by a macrocyclic wheel component (Ma and Tian, 2010; Guo et al., 2020). Conventionally, the axle component contains two bulky end groups as stoppers to protect the wheel component from dethreading. In addition, when the main axle has two or more different recognition stations, the macrocyclic wheel component can move reversibly along the axle chain between different binding sites under external stimuli, and thus a molecular shuttle is formed. For instance, for a two-station molecular shuttle, when two binding sites are the same, the wheel component will move along the axle between these two stations spontaneously, leading to a degenerate molecular shuttle. While two binding sites are different, depending on the binding affinities between binding sites and wheel component, two different equilibrating co-conformations would co-existed. In this case, by using specific stimuli to modulate the differences in binding affinities, the controllable shuttling of the wheel component between two binding sites would be feasible, resulting in a non-degenerate controllable molecular shuttle. Since the Stoddart group reported the first degenerate molecular shuttle in 1991 (Anelli et al., 1991) and the first non-degenerate controllable molecular shuttle in 1994 (Bissell et al., 1994), the investigations of molecular shuttles have been widely carried out. Research mainly focuses on two aspects: how to effectively control the shuttling processes and how to realize practical applications of molecular shuttles (Lee et al., 2009; Young et al., 2014; Corra et al., 2019; Wu et al., 2020).

Several methods, such as pH changes, redox stimuli, cation or anion bindings, solvent changes, and light irradiation have been envisioned to trigger motions in molecular shuttles, making them excellent candidates as novel molecular machines (Xue et al., 2015; Kassem et al., 2017; Wang Q. et al., 2018). Among these external stimuli, light is extraordinarily attractive since it offers several distinct advantages: light is directional and can be induced to a precise area, many photochemical reactions do not generate any byproducts, the wavelength could be well modulated to affect only certain functional groups, and remote regulation can be easily realized (Balzani et al., 2009; Silvi et al., 2011). The light irradiation could induce changes in molecular skeleton structures, initiate photochemical reactions, or trigger photoinduced electron transfer in specific systems, and thereby drives the macrocyclic wheels to shuttle between different binding sites (Berná et al., 2005; Chatterjee et al., 2006; Raiteri et al., 2008). Meanwhile, the reversible shuttling movements of macrocyclic wheels can lead to remarkable changes in the chemical microenvironment and modify the electrical or photophysical properties of rotaxanes, which can be exploited in optical information storage, catalysis, drug delivery, ion transport, and so on.

Light-driven molecular shuttles have developed rapidly in the last decades, and they play pivotal roles in more fields. In recent years, several reviews on photoactivated artificial molecular machines have been demonstrated (Bléger and Hecht, 2015; Qu et al., 2015; Wang et al., 2018a; Yu S. et al., 2019; Groppi et al., 2019; Baroncini et al., 2020; Corra et al., 2020). However, we find that the contents of light-driven molecular shuttles are only sporadically distributed. Considering the important contribution of light-driven molecular shuttles to the studies of supramolecular systems particularly molecular machines, this review systematically summarizes the common light-driven molecular shuttles according to different operating mechanisms and briefly introduces the practical applications based on them. Considering that there have already been literatures which reviewed the early research results (Ma et al., 2009; Silvi et al., 2009), more attention is mainly focused on the literature after 2010.

## Light-Driven Molecular Shuttles Based on Different Mechanisms

### Photoinduced *Trans-Cis* Isomerization

#### *Trans−Cis* Isomerization of N=N Double Bond

Azobenzene (AB) derivatives have been widely investigated as photoresponsive compounds (Chen et al., 2020). Their configurations can be changed between *E* and *Z* forms corresponding to visible light and UV irradiations, which can be utilized to construct light-responsive molecular shuttles. With AB as a photoresponsive unit in the main axle, the host macrocyclic molecules are mostly α-cyclodextrin (α-CD) in early studies (Murakami et al., 2005; Qu et al., 2005; Coskun et al., 2009). Developing other macrocycles to modify host-guest interactions is of great importance. During the past few years, several macrocycles such as cucurbituril and pillar [6]arene have been employed as wheel components for the construction of novel AB-based molecular shuttles, which would further expand their practical applications.

**FIGURE 1:**
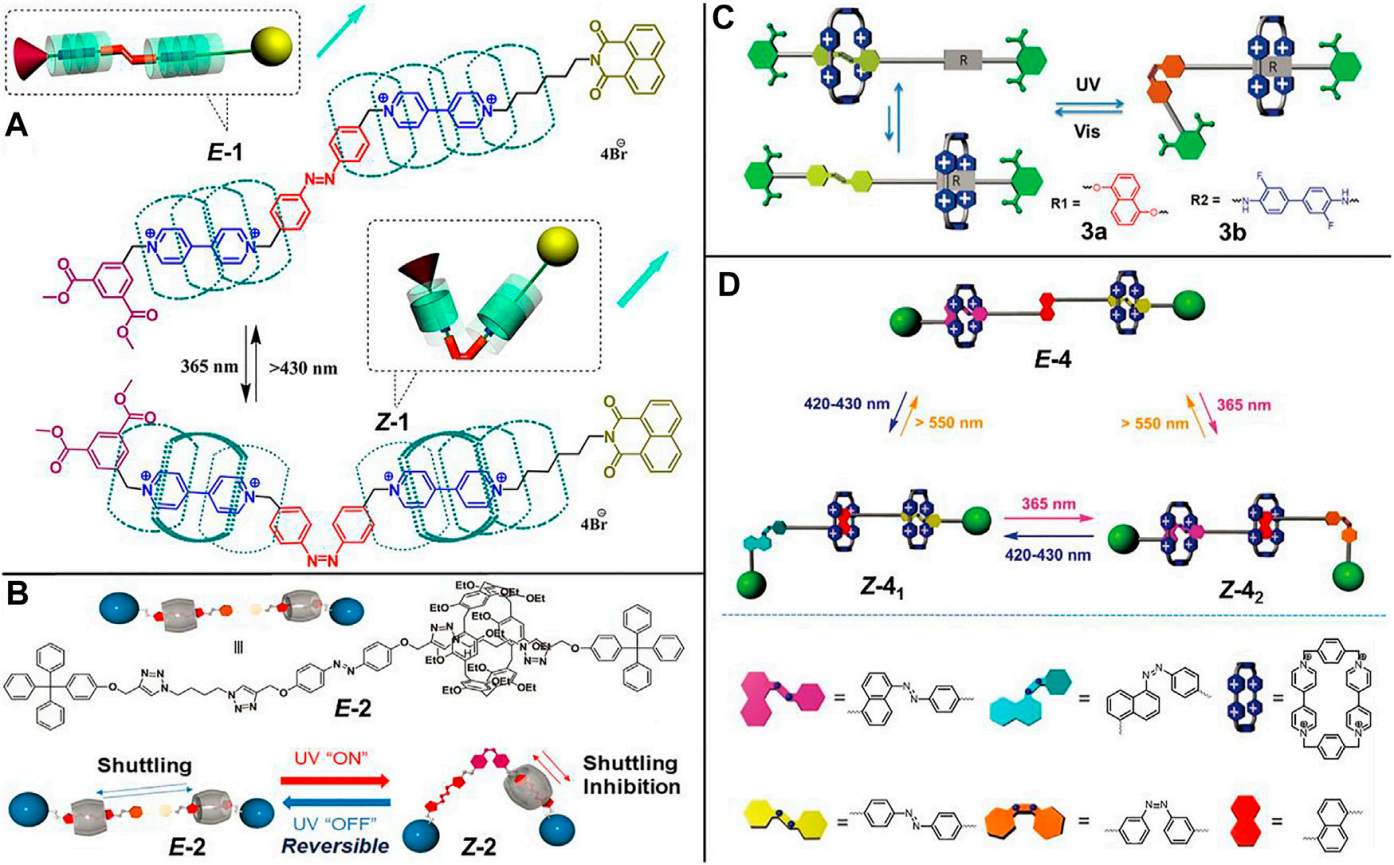
Chemical structures of molecular shuttles based on azobenzene units and schematic representations of their light-driven shuttling processes. **(A)** [3]Rotaxane **1**. Adapted with permission from Zhu et al. (2012). Copyright (2012) American Chemical Society. **(B)** [2]Rotaxane **2**. Adapted with permission from Ogoshi et al. (2020). Copyright (2020) Royal Society of Chemistry. **(C)** [2]Rotaxanes **3a** and **3b**. Adapted with permission from Zhan et al. (2016). Copyright (2016) Royal Society of Chemistry. **(D)** Tristable [3]rotaxane **4**. Adapted with permission from Li et al. (2021). Copyright (2021) Royal Society of Chemistry.

Zhu et al. (2012) reported a light-driven molecular shuttle based on cucurbit [7]uril ([3]rotaxane **1**, Figure 1A). The [3]rotaxane **1** contains two cucurbit [7]uril macrocycles and a dumbbell component composed of an *E*-AB unit as well as two viologen units as two recognition sites. Under ***E*-1** isomer state (corresponding to 430 nm irradiation), the shuttling scope of two cucurbit [7]uril rings was expanded from the viologen subunits to the phenyl subunits of AB unit. With 365 nm light irradiation, the shuttling equilibrium distribution in ***Z*-1** was diverted from the AB units to the viologen units arising from the steric hindrance after the *trans-to-cis* photoisomerization of the AB unit.

Ogoshi et al. (2020) designed a light-operated molecular shuttle ([2]rotaxane **2**, Figure 1B) based on pillar [6]arene. In their study, the AB unit served as a photo-gate to modulate the shuttling behavior of pillar [6]arene macrocycle. In the *E*-**2** form, the pillar [6]arene macrocycle could shuttle freely and reversibly between two recognition sites, while slow or no shuttling occurred in the Z-isomer. The above shuttling processes could be qualitatively characterized by ^1^H-NMR spectrum and spectral methods, such as UV-vis absorption spectrum and fluorescence spectrum.

Zhan et al. (2016) employed the famous “blue box”, namely cyclobis (paraquat-p-phenylene) (CBPQT^4+^) macrocycle, as an acceptor host unit to construct an AB-based molecular shuttle (Figure 1C). Initially, they designed and synthesized two [2]rotaxanes **3a** and **3b** (Figure 1C), which were composed of an AB subunit and a 1,5-dioxynaphthalene (DNP, for **3a**) subunit or a 3,3′-difluorobenzidine (DFBZ, for **3b**) subunit as donor recognition stations, respectively. The CBPQT^4+^ ring could be modulated to be trapped at the AB subunit or the DNP subunit (or the DFBZ subunit) upon the photoinduced *trans-cis* isomerization of the AB unit. Importantly, when the CBPQT^4+^ ring was residing on the DNP or the DFBZ site, the charge-transfer (CT) absorption bands of DNP⊂CBPQT^4+^ (*λ*_max_ = 560 nm) or DFBZ⊂CBPQT_4+_ (*λ*_max_ = 650 nm) complex were outside of the photochromically active absorption bands (365 and 420 nm), making it be potentially meaningful for the construction of non-destructive readout systems in optical information storage. Based on these results, they further synthesized a [3]rotaxane **4** (Figure 1D), in which the AB subunit and the naphthalene-derived azobenzene (NP-AB) subunit were linked by a DNP subunit (Li et al., 2021). The *trans-to-cis* isomerizations of AB (*λ* = 365 nm) and NP-AB (*λ* = 420–430 nm) units correspond to lights of different characteristic wavelengths, and thus [3]rotaxane **4** is a unique orthogonal photoresponsive tristable molecular shuttle, which is of great importance for realizing complicated functionality and advanced applications.

#### *Trans−Cis* Isomerization of C=C Double Bond

**FIGURE 2:**
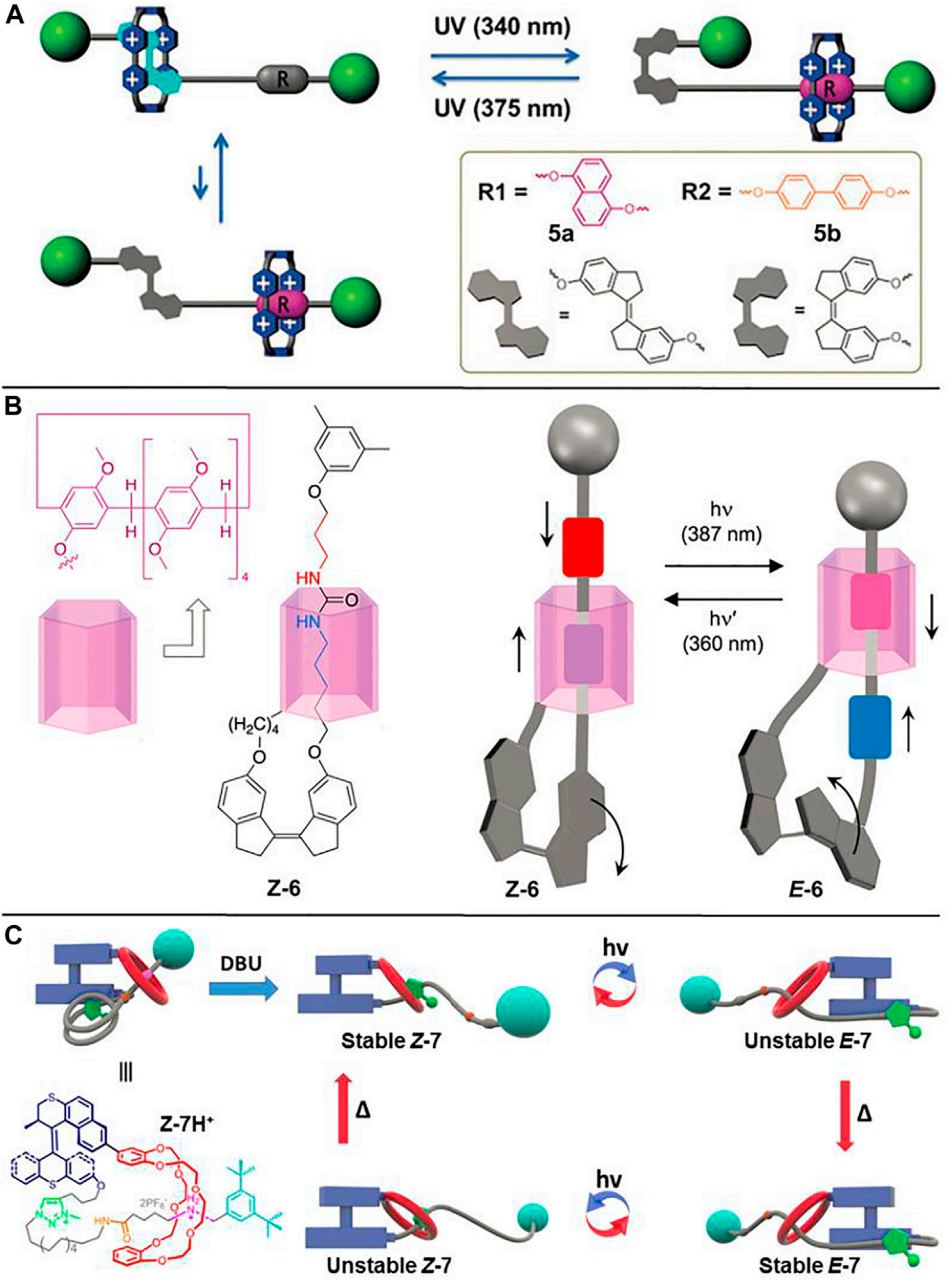
Chemical structures of molecular shuttles by virtue of a C=C double bond and schematic illustrations of their light-driven shuttling processes. **(A)** [2]Rotaxanes **5a** and **5b**. Adapted with permission from Zhan et al. (2018). Copyright (2018) Royal Society of Chemistry. **(B)** [1]Rotaxane **6**. Adapted with permission from Wang et al. (2018). Copyright (2018) Royal Society of Chemistry. **(C)** [1]Rotaxane **7**. Adapted with permission from Yu et al. (2019). Copyright (2019) American Chemical Society.

Attributed to the synthetic convenience and facile regulation of molecular recognition properties, the AB-based rotaxanes described above are the most studied light-driven molecular shuttles. However, due to the visible light responsive characteristic of the AB unit, the corresponding *Z*-isomer usually fades once exposed to visible light that exists widely in natural and artificial environments, thus limiting their applications in data storage, optical displays, etc. To address the problem of the visible light responsive property of the AB unit, the development of photofunctional groups that respond to wavelengths outside the visible light window is of great importance. Zhan et al. (2018) reported two bidirectional photoswitchable molecular shuttles ([2]rotaxanes **5a** and **5b**, Figure 2A) with long-term visible light stability with the aid of a stiff stilbene (SB) unit based on the *trans−cis* isomerization of the C=C double bond.

As displayed in Figure 2A, the SB unit is solely UV light sensitive, which can perform *trans–cis* photoisomerization upon distinguished wavelength irradiations. The SB unit, DNP (for **5a**) or biphenyl (BP) (for **5b**) units were incorporated into the axle components of [2]rotaxanes as different recognition stations, while the CBPQT^4+^ macrocycle was selected as a wheel component. Upon irradiation with UV light (*λ* = 340 nm), the SB unit underwent *trans-to-cis* photoisomerization, driving the CBPQT^4+^ macrocycle to shuttle from the SB unit to the DNP unit (45%) or the BP unit (48%). When irradiated by another UV light (*λ* = 375 nm), the CBPQT^4+^ ring moved back to the *E*-SB unit owing to the *cis-to-trans* isomerization of the SB unit (92 and 91% for **5a** and **5b**, respectively). Further time lapse absorption spectra indicated that both ***Z*-5a** and ***Z*-5b** could maintain long-term stability when their UV light irradiated solutions were exposed to visible light environment for several days.

The above-mentioned examples of photoisomerization of N=N or C=C based molecular shuttles are mostly exploited steric hindrances to block the translation motion of the macrocyclic wheel. Wang Y. et al. (2018) demonstrated another alternative method for gaining light-driven translation of one molecular subunit relative to the other one based on a [1]rotaxane **6** (Figure 2B). As shown in the figure, the pillar [5]arene macrocycle was connected with the SB unit by a proper linker in [1]rotaxane **6**, so the SB unit could serve as a functional group to induce the movement of pillar [5]arene wheel and concomitantly act as a stopper to prevent the macrocycle from dethreading. The movement of pillar [5]arene unit arises from the contraction or elongation of the SB unit upon its *trans-cis* photoisomerization under distinguishable UV light irradiations.

Based on similar molecular design, Yu J.-J. et al. (2019) also demonstrated different motion modes could be combined in an MIM ([1]rotaxane **7**, Figure 2C), and successfully achieved a four-step operating cycle. When irradiated with different UV lights, the *trans-cis* photoisomerization of the C=C double bond in the S−S molecular motor rendered the dibenzo-24-crown-8 (DB24C8) ring to shuttle between benzylalkylammonium hexafluorophosphate (BAA) and N-methyltriazolium hexafluorophosphate (MTA) recognition stations, giving unstable ***E*-7** or unstable ***Z*-7**. The subsequent heating processes could induce helix inversion and convert the unstable species to the corresponding stable states.

The examples of **6** and **7** clearly provide evidence that a molecular motor can work against noncovalent interactions in MIMs and the movement of the rotaxane can be induced by a transmission mechanism, which gives deeper insights into the operating capacity in more complicated molecular machines.

#### *Trans−Cis* Isomerization of C=N Double Bond

**FIGURE 3:**
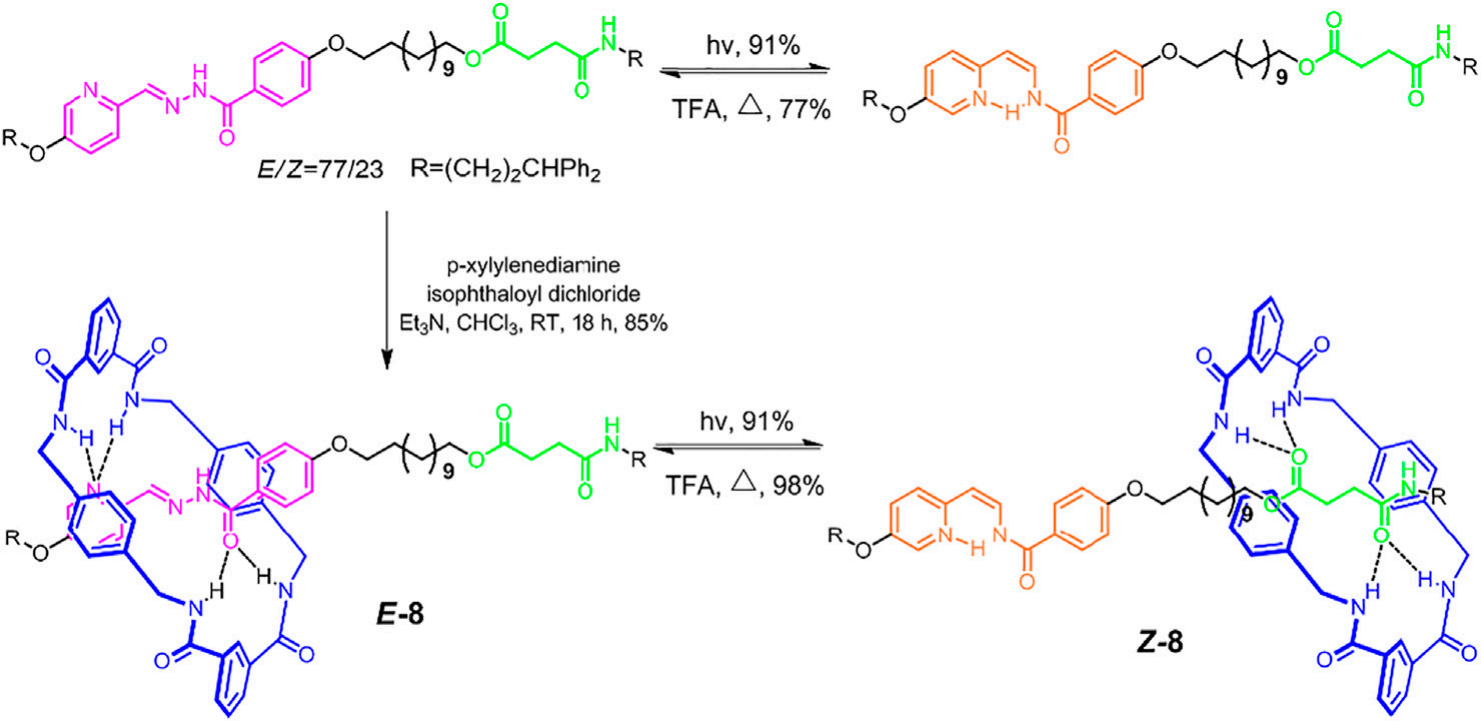
Synthesis and light-driven switching processes of axle and molecular shuttle [2]rotaxane **8**. Adapted with permission from Leigh et al. (2017). Copyright (2017) American Chemical Society.

Usually, the light-driven molecular shuttles would meet several problems, such as low positional integrity, only stable under special environment, and poor switching fidelity. Benefiting from the photoinduced *trans−cis* isomerization of the C=N double bond, Leigh et al. (2017) reported a molecular shuttle ([2]rotaxane **8**, Figure 3) that could overcome all these problems. The highly directional hydrogen bonds between benzylic amide macrocycle and recognition sites (pyridyl-acyl hydrazone unit or succinic amide ester unit) were the main driving force. The pyridyl-acyl hydrazone unit could present *trans-cis* isomerization with extra light or thermal stimuli in high efficiency. In the ***Z*-8** form (under 312 nm UV light stimulus), the pyridyl and hydrazone units would form a six-membered intramolecular hydrogen bond system. Owing to the significantly different binding affinities of *E/Z* forms of pyridyl-acyl hydrazone for a wheel component, the macrocyclic wheel could reside on the thread with high positional integrity (>95%). The isomerization efficiencies of both ***Z*-8** and ***E*-8** exceeded 90%, which represented one of the highest switching fidelities reported. Notably, in contrast to the *trans-cis* isomerizations of N=N and C=C double bonds, both of which could be solely operated by light with different wavelengths, the *cis−to−trans* isomerization of the abovementioned C=N double bond needs an external addition of strong polar solvent to break hydrogen bonds, which might have an adverse effect on multiple cycle operations.

### Photochemical Reactions

For some specific functional groups, they can undergo photochemical bond-cleavages upon light irradiation and return to original states with different stimuli, such as light, heat, pH, and so on (Baroncini et al., 2020). These attractive properties are quite suitable for the construction of light-driven molecular shuttles. The pioneering works were from Abraham’s group who have successfully achieved light-driven molecular shuttles by means of the photoheterolysis of methoxy-substituted cycloheptatriene (Abraham et al., 2004) and methoxy-substituted acridane (Abraham et al., 2007).

**FIGURE 4:**
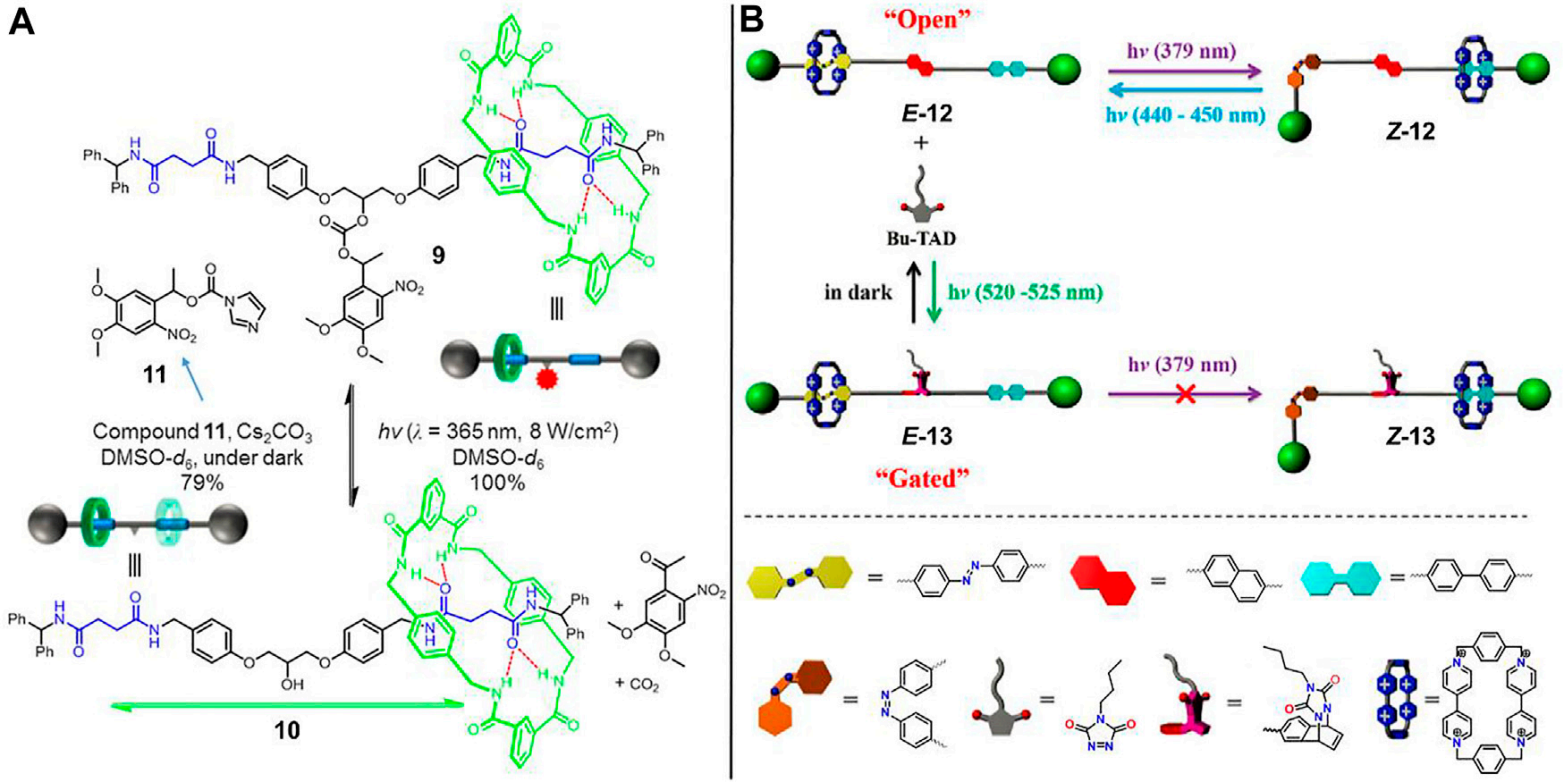
Light-driven molecular shuttles based on photochemical reactions. **(A)** Photoheterolysis and rebonding reactions of [2]rotaxanes **9** and **10**. Adapted with permission from Gao et al. (2017). Copyright (2017) American Chemical Society. **(B)** Reversible cycloaddition reactions of [2]rotaxanes **12** and **13**. Adapted with permission from Yang et al. (2021). Copyright (2021) Elsevier.

Inspired by the photochemical bond-cleavage strategy, Gao et al. (2017) successfully triggered a novel molecular shuttle ([2]rotaxane **9**, Figure 4A) by a photoactive carbonate ester moiety. As displayed in the figure, the symmetrical [2]rotaxane **9** has two succinimide units as identical recognition stations. A photoresponsive bulk barrier was chemically bonded into the middle of the axle to prevent the dynamic shuttling of the wheel component, leading to a “gated” state where the macrocycle has a definite location at one of two recognition sites. Upon irradiation of UV light, the bulk barrier was removed by the complete photoheterolysis of the carbonate ester, resulting in the subsequent recovery of fast shuttling movement of the macrocycle between two identical recognition sites, which was referred to as the “open” state ([2]rotaxane **10**). The reverse open-to-gated process could be modulated via a reconnecting of the trigger into rotaxane by virtue of molecule **11**.

Yang et al. (2021) reported a molecular shuttle ([2]rotaxane **12**, Figure 4B) modulated by a photoinduced cycloaddition reaction. As illustrated in Figure 4B, the axle of rotaxane **12** bears a BP unit and a photoswitchable AB unit as different recognition stations, which are bridged via a naphthalene (NAP) subunit. In the “open” state, the CBPQT^4+^ macrocycle was initially trapped at the AB unit, and it could be reversibly triggered to shuttle between two recognition stations by the *trans-cis* isomerization of the AB unit upon UV/visible light. The free shuttling motion would be predominantly blocked by the introduction of steric hindrance generating from the green-light induced cycloaddition reaction between the NAP subunit and triazolinedione. Upon staying in the dark, the cycloaddition product autonomously underwent disassociation, contributing to the recovery of light-driven shuttling capability of the macrocycle.

These above-discussed examples clearly indicate that the construction of molecular shuttles via photochemical reactions can provide novel approaches to enhance the shuttling controllability of rotaxane. Notably, there are still some key issues that need to be addressed for a photochemical reaction strategy. For instance, the addition reaction is incomplete (i.e., 79% for **9**) because it is a bimolecular process, while the reverse bond-cleavage process is very efficient due to it is a unimolecular reaction. The employment of more efficient and reversible photochemical reactions will be in great demand for the further expansion of this novel type of light-driven molecular shuttles.

### Photoinduced Electron Transfer

Electron transfer is essentially a redox reaction and plays a key role in many important life processes. In addition to be driven by redox agents, many electron transfer processes could be actuated photochemically, which have been successfully demonstrated for light-driven molecular shuttles.

**FIGURE 5:**
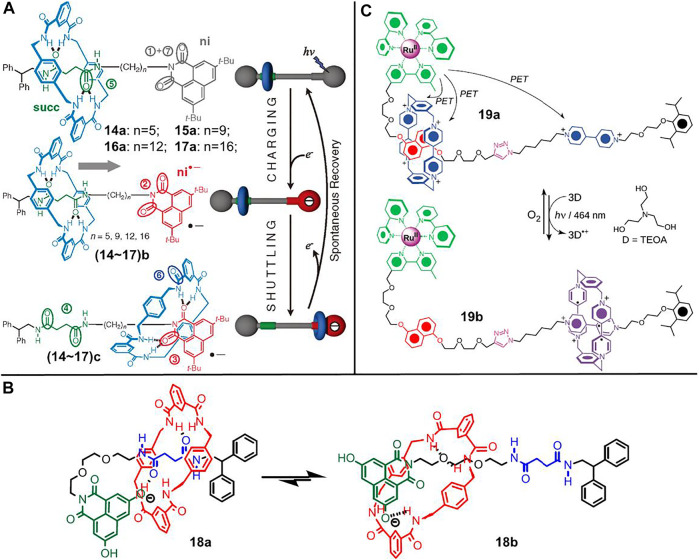
**(A)** Chemical structures of [2]rotaxanes **14∼17** and their light-driven shuttling behaviors between different states. Adapted with permission from Panman et al. (2010). Copyright (2010) AAAS Science. **(B)** Chemical structure of [2]rotaxane **18** and the proposed “harpooning” mechanism for the shuttling process. Adapted with permission from Kumpulainen et al. (2019). Copyright (2019) American Chemical Society. **(C)** Chemical structure of [2]rotaxane **19** and the photoswitchable feature based on radical–radical interactions. Adapted with permission from Li et al. (2011). Copyright (2011) Wiley-VCH Verlag GmbH & Co. KGaA.

Brouwer et al. (2001) creatively reported a benzylic amide-based molecular shuttle ([2]rotaxanes **16**, Figure 5A) which responded to photochemical stimuli. The molecular shuttle **16** consists of a benzylic amide ring interlocked onto an axle that bears a succinamide (**succ**) unit and a 3,6-di-tert-butyl-1,8-naphthalimide (**ni**) unit as two potential hydrogen bonding sites. The benzylic amide ring was first trapped at the **succ** station due to the bifurcated hydrogen bonds between the isophthalamide groups in the wheel component and the two amide carbonyls in the **succ** unit. After photoexcitation, the **ni** chromophore underwent intersystem crossing to its triplet (T) state, and subsequently formed an **ni** radical anion when reducted by an electron donor reagent (DABCO). The **ni** radical anion had an enhanced H-bond–accepting affinity for the benzylic amide ring, rendering the ring to move from the **succ** site to the **ni** site. The **ni** radical anion could be reversibly converted to the neutral state upon recombination with the DABCO radical cation, making the system return to the ground state and the amide macrocycle shuttle back to the **succ** site.

The shuttle processes were originally confirmed by time resolution transient absorption spectroscopy and ^1^H-NMR spectroscopy, both of which did not allow for the inspection of singlet state with short lifetime. Subsequently, they developed an effective method to gain more insight into the shuttling mechanism using time-resolved vibrational spectroscopy by virtue of the distinguishable CO-stretch frequencies of carbonyl groups in different states (Panman et al., 2010; Panman et al., 2012). The shuttling of macrocycle was found to undergo two steps: photothermally driven escaping from the initial site (**succ**), followed by fast movement along the axle ending either at the **succ** unit or the **ni** unit. Through varying the length of the alkyl spacer (**14∼17**, *n* = 5, 9, 12, 16, Figure 5A), the rapid motion was further confirmed as a biased one-dimensional random walk mechanism (Panman et al., 2010).

Besides the alkyl spacer, the effect of solvent was also systematically investigated. In particular, the experiments were performed in viscous polymer solutions, and the photoinduced shuttling rates were found to be slowed down in polymer solutions (Jagesar et al., 2009). The retardation effect could be reasonably explained by a hydrodynamic scaling model, which was correlated to the presence of more entanglements of polymer chains in viscous solutions. In 2013, water was found to be acting as a molecular lubrication to significantly accelerate the shuttling rates, which was ascribed to the 3D hydrogen-bond network formed between the shuttling parts of molecular shuttle (Panman et al., 2013).

In 2019, the effect of axle and stopper on the shuttling rates was proved (Kumpulainen et al., 2019). As shown in Figure 5B, in comparison to **14∼17**, the alkyl spacer and 3,6-di-tert-butyl-1,8-naphthalimide were replaced by a polyether segment (PE, C_6_O_2_) and a novel 3,6-dihydroxy-1,8-naphthalimide (dOH-NI) unit in [2]rotaxane **18**. Both PE and dOH-NI units were proved to accelerate the shuttling rates, but the operating mechanisms were different. The acceleration of the PE unit was ascribed to the decrease of activation energy because of an early transition state, in which the benzylic amide ring was partly bonded to the ether functional group. The acceleration of the dOH-NI unit could be interpretated by a harpooning mechanism. In other words, the transition state involved a folded conformation on account of the hydrogen bonding interactions between macrocycle and dOH-NI unit.

Most of the above-mentioned molecular shuttles were performed in organic solvents. In consideration to gain more insight into the functions of biological molecular machines which are mainly operated in aqueous media, molecular shuttles operated in aqueous conditions are attractive. Li et al. (2011) demonstrated a light-driven molecular shuttle ([2]rotaxanes **19**, Figure 5C) driven by radical–radical interactions in aqueous solution. The [2]rotaxane **19** contains a CBPQT^4+^ macrocycle and an axle bearing a DNP unit and a 4,4′-dialkylbipyridinium (BIPY^2+^) unit as distinct recognition sites. Meanwhile, the dumbbell component has an [Ru (bpy)_3_]^2+^ complex as one stopper. The CBPQT^4+^ macrocycle initially encircled the π-electron-rich DNP unit. Upon irradiation with visible light, all three BIPY^2+^ units were reducted to radical cation states (BIPY^•+^) by the excited [Ru (bpy)_3_]^2+^ complex, after which the Ru element was reducted from Ru^III^ state to Ru^II^ state by a sacrificial reagent, such as triethanolamine (TEOA). The resultant diradical dicationic CBPQT^2(•+)^ underwent movement to encircle the BIPY^•+^ unit owing to the stabilizing radical–radical interactions between the CBPQT^2(•+)^ unit and the BIPY^•+^ unit, as well as the loss of D–A interactions between the DNP station and the CBPQT^2(•+)^ macrocycle. When the BIPY^•+^ units were oxidized by external oxygen, the recovery of D–A interactions and the Coulombic repulsion between CBPQT^4+^ macrocycle and BIPY^2+^ unit rendered the ring to shuttle back to the DNP unit. All the processes could be operated in aqueous solution and were found to be reproducible with no evidence of obvious decomposition detected over five operating cycles.

### Light-Driven Non-Photoresponsive Molecular Shuttles

**FIGURE 6:**
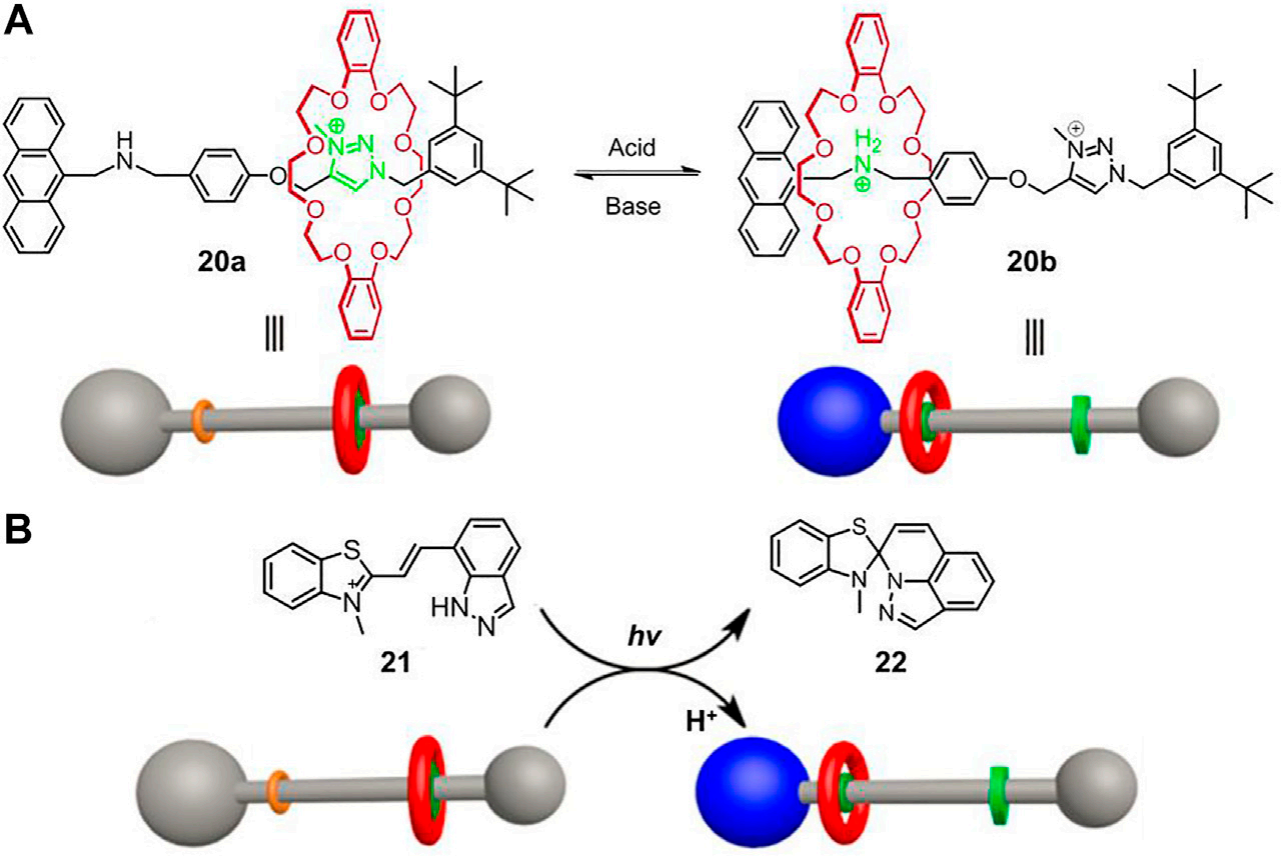
**(A)** Chemical structure and schematic diagram of acid−base based molecular shuttle [2]rotaxane **20**; **(B)** the light-driven shuttling of non-photoresponsive **20** by a photoacid **21**. Adapted with permission from Yang et al. (2017). Copyright (2017) American Chemical Society.

Light-controlled switching of a non-photoresponsive rotaxane will be undoubtedly challenging since axle component and wheel component are both non-photoactive. However, light can change the microenvironments around rotaxanes, leading to an indirect switching of molecular shuttle. Yang et al. (2017) reported the first example of light-controlled operation of a non-photoresponsive molecular shuttle ([2]rotaxane **20**, Figure 6) stemming from a photoinduced proton-transfer (PIPT) strategy.

As shown in Figure 6A, [2]rotaxane **20** is essentially an acid/base-switchable molecular shuttle, in which the dibenzo-24-crown-8 (DB24C8) macrocycle can be controllably switched between ammonium station and *N*-methyltriazolium station, corresponding to a protonated state and a deprotonated state, respectively. Compound **21** is an indazole-based photoacid that could behave as photoinduced acidity with good reversibility. After irradiation of a 1.2:1 mixture of **21** and **20a** in DMSO-d_6_ solution for 5 min, **21** was completely converted to **22** and meanwhile released a proton to induce the shuttling movement of the macrocycle (Figure 6B), which was confirmed by fluorescent and NMR spectra. However, the inverse process was not implemented, which was presumably due to the strong binding affinity of **20b** for proton.

**FIGURE 7:**
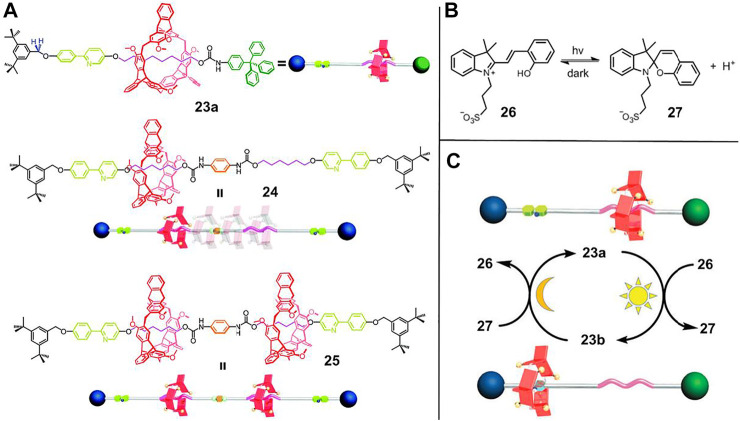
**(A)** Chemical structures of rotaxanes **23**, **24**, and **25**; **(B)** principle of photogenerated acid; **(C)** the light-driven motion of [2]rotaxane **23** by PIPT strategy. Adapted with permission from Shi et al. (2018). Copyright (2018) Royal Society of Chemistry.

To improve the reversibility of light-driven processes, several factors, such as the host-guest interactions between macrocycle and recognition sites, the proton binding capacity of recognition sites and the acidity of photoactive small molecule must be compatible. Shi et al. (2018) verified a reversible regulation of the PIPT process through rational molecular design. As illustrated in Figure 7, in [2]rotaxane **23a**, 2,6-helic [6]arene macrocycle was selected as wheel component while alkyl group and 2-phenylpyridium served as two recognition stations. Besides, molecule **26** was chosen as photoacid. Initially, the macrocycle was bound to the alkyl group due to the C–H···π interactions in solution (CD_3_OD/CD_2_Cl_2_ = 11:5). After irradiation with 420 nm or sunlight for 5 min, the photoacid **26** could protonate the pyridine unit of **23a** and therefore induce the macrocycle to shuttle to the pyridium site. Thanks to the appropriate host-guest interactions and proton binding capability of the pyridium site, the macrocycle moved back to the alkyl moiety after the solution was treated under dark conditions for 3.5 h. Therefore, the system enabled a reversible protonation and deprotonation cycle which was implemented by alternating the irradiation and dark conditions. Notably, the light-driven process of **23** could be operated more than 50 times with perfect reproducibility without any waste products. Apart from the switching motion of **23**, the oscillation motion of **24** and the palindromic motion of **25** could also be accomplished by the PIPT strategy.

**FIGURE 8:**
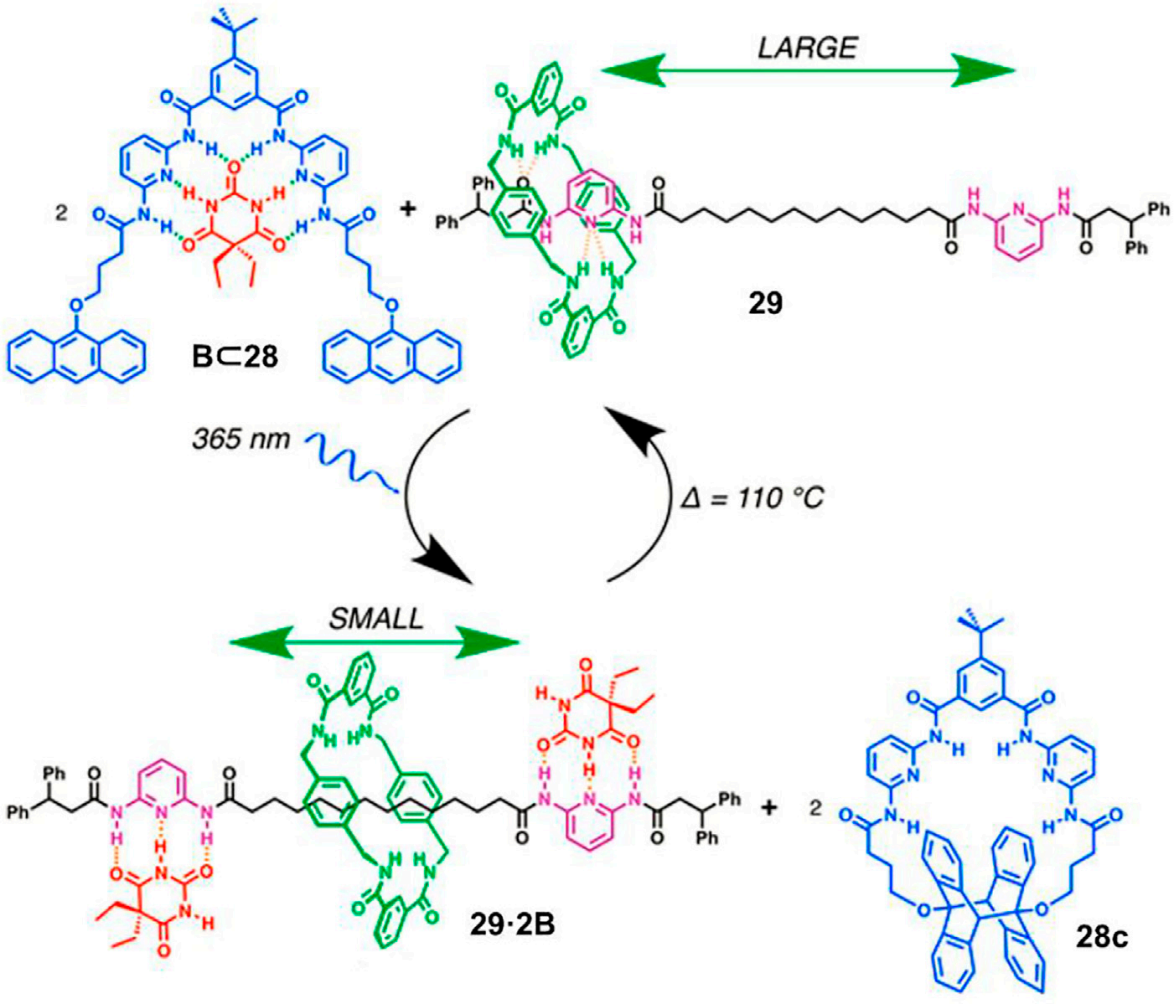
Schematic representation of the photoregulated translational movement of a macrocycle in [2]rotaxane **29** via compound **28**. Adapted with permission from Tron et al. (2017). Copyright (2017) American Chemical Society.

The aforementioned PIPT process involves a photoacid intermediate, which is unfavorable for pH-sensitive substrate. Tron et al. (2017) proved the regulation could be operated under the neutral state by virtue of a photoinduced cyclomerization reaction of anthracene. As depicted in Figure 8, compound **28** strongly bound with barbital (B) in its open form with six hydrogen bonds in the initial state. Photoirradiation of **28** induced an intramolecular anthracene cyclomerization reaction to produce **28c**, and thus B was released and transferred to bind with two diamidopyridine recognition sites of [2]rotaxane **29** which had an affinity for B laying between **28** and **28c**. Therefore, the amplitude of macrocycle shuttling was directly restricted to a small range. The reverse process was investigated by heating the system to 110°C, leading to the retrocyclomerization of **28c** and the recovery of a large translational movement of macrocycle.

Light-driven non-photoresponsive molecular shuttles are essentially photochemical reaction processes. In comparison to the abovementioned photochemical reaction strategy, the positions of the photochemical reactions are different. The photochemical reactions discussed in this section occur on other substrates, rather than rotaxanes, thus resulting in an indirect approach. However, these two approaches also have common characteristics: photochemical reactions involving the breaking of old bonds and the formation of new ones. Therefore, the whole process consists of several unimolecular reactions and bimolecular reactions. Different reactions will vary greatly in reaction rates, and thus some processes require additional operations, such as prolonging reaction time or heating.

## Applications

### Optical Information Storage

**FIGURE 9:**
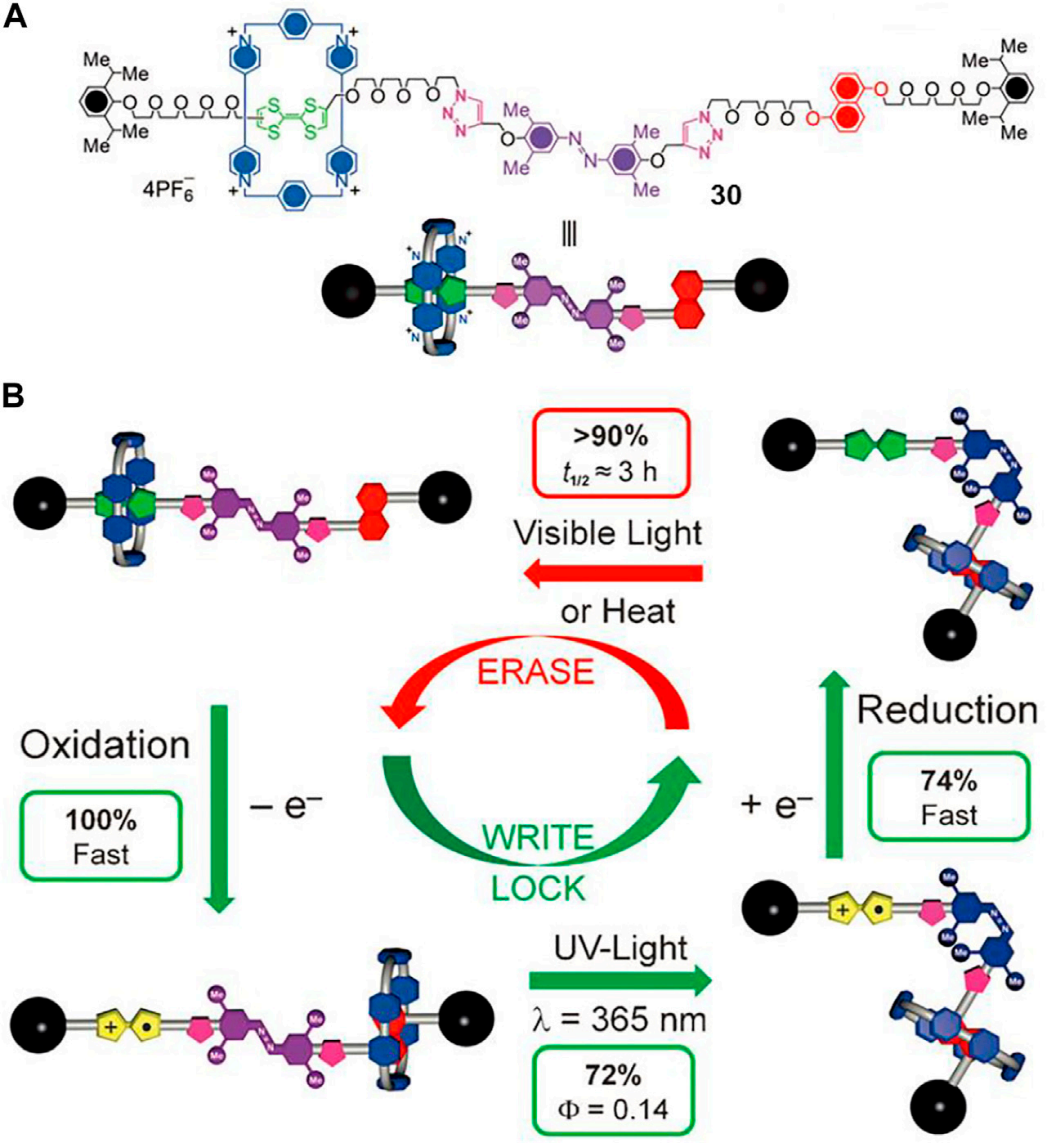
**(A)** Chemical structure of [2]rotaxane **30**; **(B)** memory switching cycle of [2]rotaxane **30** ^4+^ triggered by chemical and photochemical methods. Adapted with permission from Avellini et al. (2012). Copyright (2012) Wiley-VCH Verlag GmbH & Co. KGaA.

Molecular shuttles can be switched between different bistable or multi-states in response to external stimuli, which is similar to contemporary integrated systems and devices, so light-driven molecular shuttles can be potentially utilized for optical information storage (Zhang et al., 2012). Apart from thermodynamically different stable states, a convenient, efficient, and reversible shuttling kinetics is also of great importance. Avellini et al. (2012) designed and synthesized a multi-stimuli responsive molecular shuttle ([2]rotaxane **30**, Figure 9A) to investigate its photoinduced memory effect, which could be thermodynamically transformed between two states and kinetically controlled by photochemical means.

The [2]rotaxane **30** ^4+^ contains a CBPQT^4+^ macrocycle as a wheel component, a tetrathiafulvalene (TTF) unit and a DNP unit as the initial and second recognition stations, and a 3,5,3′,5′-tetramethylazobenzene (TMeAB) unit which could be switched between *Z* and *E* configurations upon light-stimuli. A “*write-lock-erase*” experiment was conducted to investigate the light- and redox-induced memory switching cycle of [2]rotaxane **30** ^4+^. As can be seen in Figure 9B, the switching cycle was composed of four sections, and detailed experimental results suggested that [2]rotaxane **30** ^4+^ could serve as a bistable memory material under reasonable kinetic control. After the oxidation writing session, the light irradiation successfully locked the data and protected the nonoxidized species from accidental writing, and then the oxidized rotaxanes could be reducted back to the initial state without losing the written information for several hours. In other words, the written data could be stored for a remarkably long time under dark conditions and room temperature until the thermal-induced *cis-to-trans* isomerization of TMeAB occurred, which was different from other thermodynamically controlled molecular shuttles in which the metastable states co-conformations usually had short lifetimes.

### Catalysis for Organic Synthesis

**FIGURE 10:**
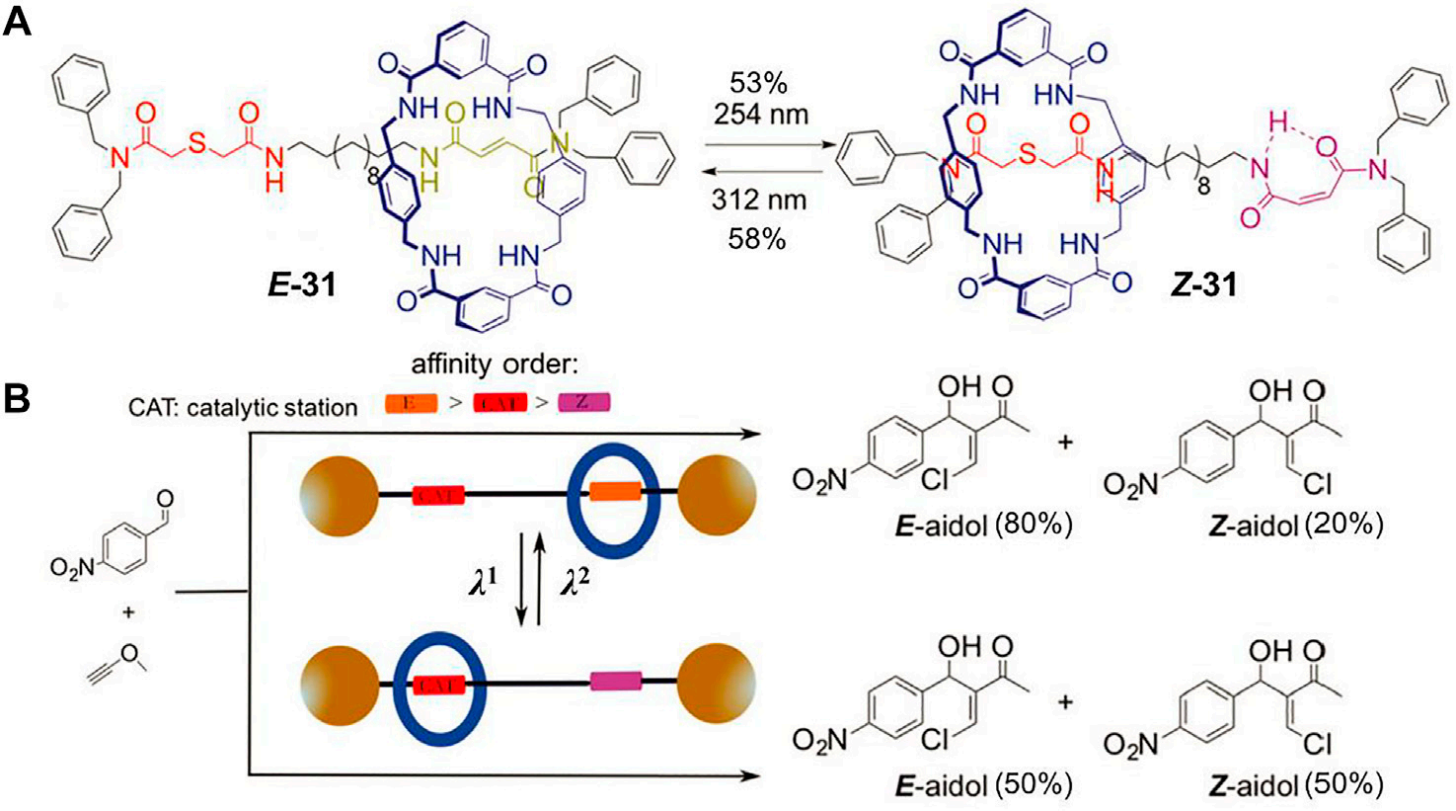
**(A)** Chemical structure and light-driven switching of [2]rotaxane **31**; **(B)** schematic representation of the photoswitchable catalyst of **31**. Adapted with permission from Martinez-Cuezva et al. (2017). Copyright (2017) Royal Society of Chemistry.

Gaining catalysts with switchable activity to simulate the enzymatically driven processes is a growing research topic nowadays. MIMs can be controllably switched between different states and thus they are promising candidates for novel switchable catalysts. Notably, pH-driven molecular shuttles have been successfully employed for controlling the catalytic activities of several particular reactions (Blanco et al., 2014a; Blanco et al., 2014b; Eichstaedt et al., 2017). In 2017, Martinez-Cuezva et al. (2017) first reported a light-driven molecular shuttle ([2]rotaxane **31**, Figure 10A) acted as a cocatalyst component for chalcogeno-Baylis–Hillman reaction.

The molecular shuttle **31** is a tetralactam-based [2]rotaxane which contains four carbonyl groups that can form hydrogen bonds with a guest molecule. One recognition site of the axle is composed of a photoactive fumaramide, and the other is a sulfur-containing thiodiglycolamide which has two carbonyl groups as hydrogen bonding acceptors and an embedded sulfur atom as catalytic active center. The [2]rotaxane **31** was utilized as cocatalyst to catalyze the Baylis–Hillman reaction in CH_2_Cl_2_ solution (Figure 10B). When exposed to UV light of 312 nm, the macrocycle was chiefly located at the fumaramide site and the thiodiglycolamide was exposed to the surrounding reaction substrate, thus the sulfur atom could reveal catalytic activity, leading to a better control of the reaction (*E/Z* = 80:20). Upon light irradiation at 254 nm, the photoisomerization of trans C=C double bond switched the fumaramide unit to its *cis* counterpart maleamide and the macrocycle was mainly positioned at the thiodiglycolamide unit owing to the intramolecular hydrogen bond. Therefore, the sulfur atom was locked inside the ring and the catalytic reaction produced a 1:1 mixture of both isomers. Notably, the reaction conversion using the two-station [2]rotaxane decreased in comparison to those obtained using noninterlocked catalysts, but this example first showed that the utilization of photoswitchable interlocked catalysts could be meaningful to control the stereochemical issue of an organic reaction.

**FIGURE 11:**
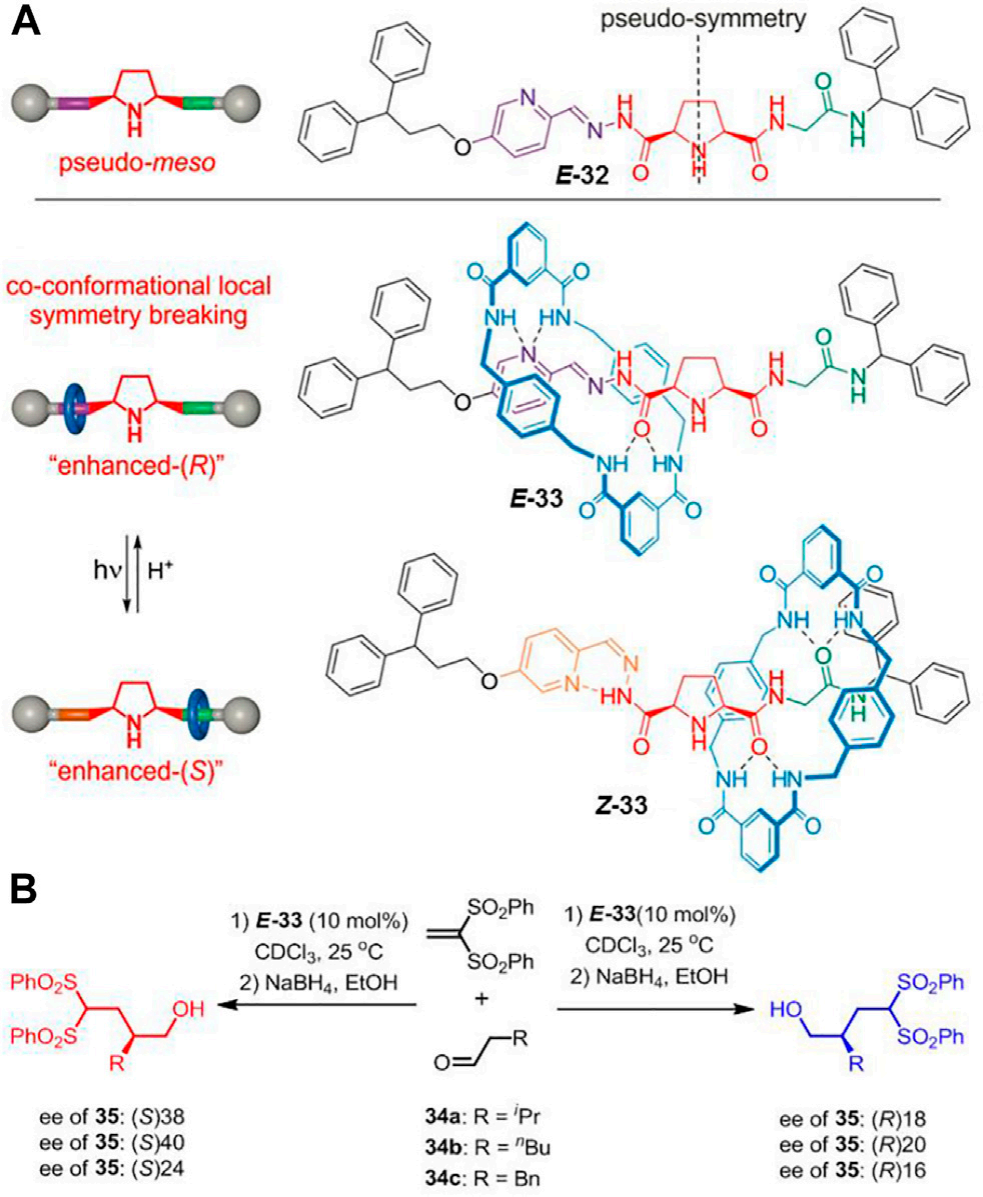
**(A)** Chemical structure of thread ***E*-32** and shuttling behavior of [2]rotaxane **33** based on the *trans–cis* photoisomerization of C=N double bond; **(B)** the asymmetric catalysis of [2]rotaxane **33** for addition reaction. Adapted with permission from Dommaschk et al. (2019). Copyright (2019) Wiley-VCH Verlag GmbH & Co. KGaA.

Apart from the strategy of locking catalytic active center, Dommaschk et al. (2019) demonstrated a local symmetry breaking approach to construct photoswitchable catalysts. As depicted in Figure 11A, the axle of [2]rotaxane **33** contains a 2,5-disubstituted pyrrolidine unit which bears a pyridyl-acyl hydrazone and a glycine amide attached to either side of it. The pyridyl-acyl hydrazone unit is photoactive since it can present *trans-cis* isomerization under different light stimuli. Initially, both ***E-*** and ***Z*-32** could be treated as pseudo-meso structures owing to the nearly symmetrical environment in the axle. Interestingly, the macrocycle would break the local symmetry of the pyrrolidine unit when the free axle was assembled into rotaxane. The [2]rotaxane presented mainly “enhanced-(R)” or “enhanced-(S)” form when the tetralactam-based macrocycle was bound to the *E*-acyl hydrazone unit or the glycine amide unit under pH or light irradiation stimuli. Both the free thread **32** and [2]rotaxane **33** were applied to catalyze the conjugate addition of aldehydes to vinyl sulfones (Figure 11B). Both ***E*-32** and ***Z*-32** did not show notable selectivity (2–14% ee of **35**), whereas the rotaxane **33** exhibited selectivity closer to 40% ee with the switching position of the macrocycle. Therefore, it strategically enabled the successful achievement of an anti-divergent catalysis with a single photoswitchable rotaxane catalyst.

### Drug Delivery

**FIGURE 12:**
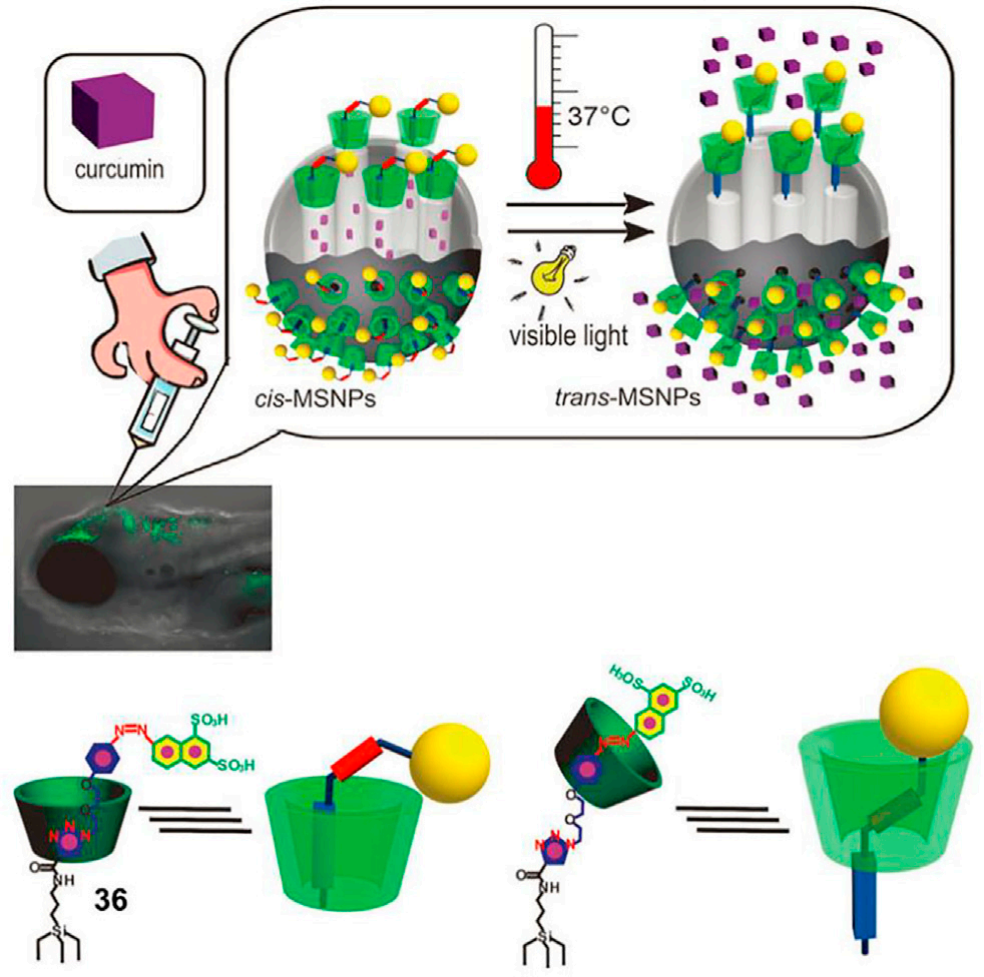
Graphic diagram of the injection of drug-containing MSNPs functionalized with [2]rotaxane **36** into zebrafish larvae for drug delivery *in vivo*. Adapted with permission from Yan et al. (2012). Copyright (2012) Wiley-VCH Verlag GmbH & Co. KGaA.

Mesoporous silica nanoparticles (MSNPs) have been applied for drug delivery, but several challenges remain to be resolved: achieving released drugs without side effects, realizing controlled release *in vivo*, and improving release efficiency (Ambrogio, et al., 2011). Yan et al. (2012) introduced a photothermal-responsive molecular shuttle to functionalize MSNPs that could successfully overcome these issues. As illustrated in Figure 12, the MSNPs were covalently attached with α-CD based [2]rotaxane **36** which contained AB and triazole/ethylene glycol units as different recognition sites, and a naphthalene disulfonate to provide solubility and as a stopper unit. The remote-controlled drug release is contributed by the shuttling movement of α-CD ring originating from the photothermal-driven *trans–cis* isomerization of an AB unit. First, the α-CD ring was trapped at the *E*-AB unit, which was away from the MSNPs surface. Subsequently, the α-CD ring shuttled to the triazole/ethylene glycol unit owing to the *trans-to-cis* isomerization of the AB unit upon UV light irradiation, and the drugs were effectively blocked into the pores of nanoparticles. The high capping efficiency was confirmed by the release of RhB-loaded MSNPs in the dark for 24 h, since a negligible amount of RhB was detected to leach. Finally, the *cis-to-trans* isomerization of an AB unit under visible light or heating stimuli made the a-CD ring shift back to the *E*-AB unit, resulting in drug delivery. Furthermore, curcumin-loaded MSNPs were successfully injected into zebrafish embryos for drug release *in vivo*, and thermal-triggered release of curcumin was shown to be more effective. These results suggest that the MSNPs decorated with photothermal-responsive molecular shuttle are excellent drug carriers for therapeutic applications.

### Ion Transport

**FIGURE 13:**
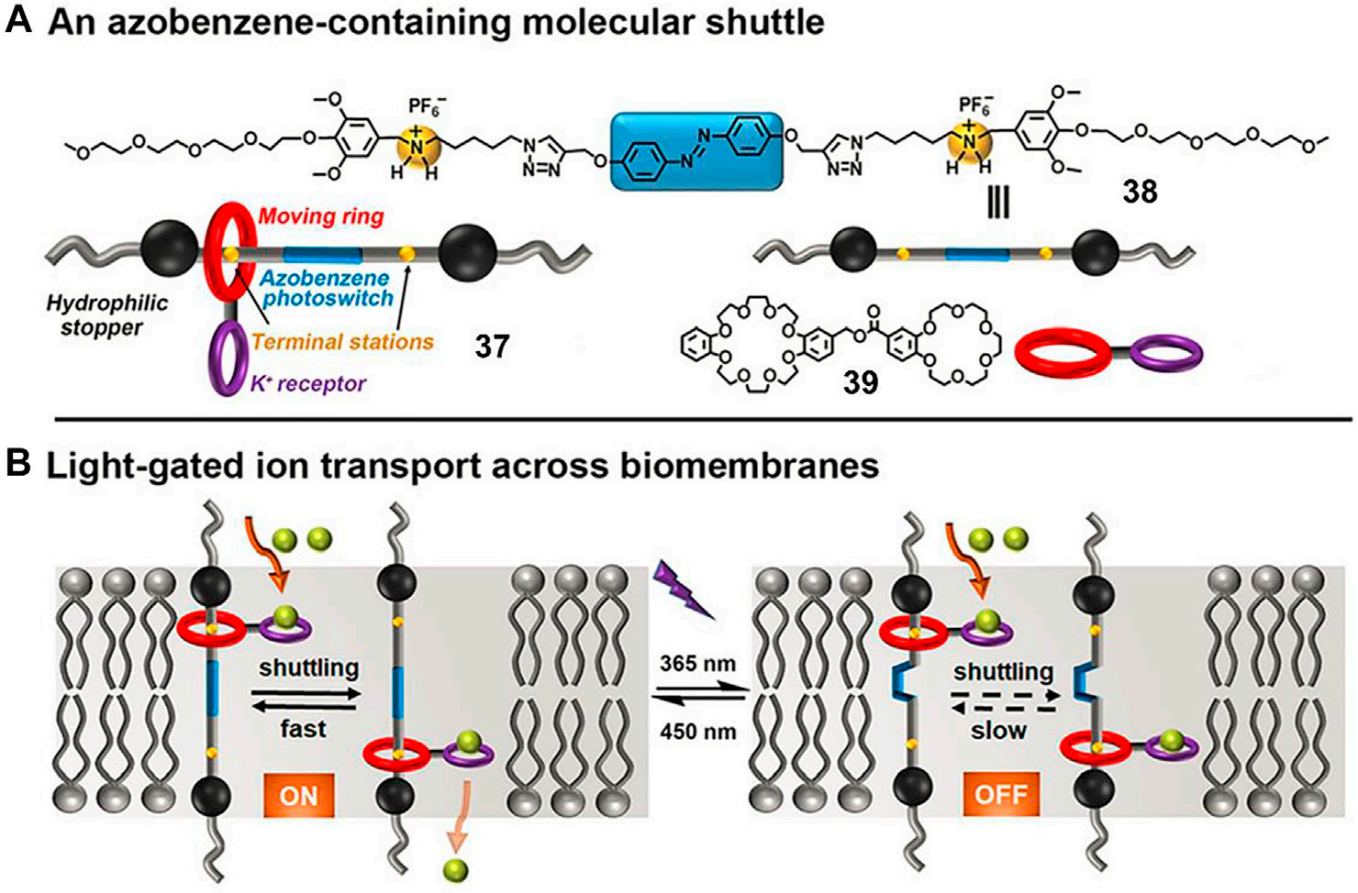
**(A)** Molecular structures of molecular shuttle **37**, amphiphilic thread **38**, and macrocycle **39**; **(B)** the possible mechanism for light-gated ion transport of **37** across biomembranes. Adapted with permission from Wang et al. (2021). Copyright (2021) Wiley-VCH Verlag GmbH & Co. KGaA.

Ion transports across the lipid bilayers play crucial roles in many biological processes. Developing artificial carriers or channels to simulate the ion transports in living organisms would improve the understanding of working mechanisms of life processes. An artificial pH-driven molecular shuttle operating in lipid bilayers for ion transport has been reported, but the gated ion transport is not implemented (Chen et al., 2018). Inspired by the working mechanism of Channelrhodopsins (ChRs), Wang et al. (2021) designed a light-driven molecular shuttle ([2]rotaxane **37**, Figure 13) with an attractive light-gated ion transport behavior.

In their study, the [2]rotaxane **37** consisted of an AB-based amphiphilic axle (**38**) with two ammonium ions as recognition sites as well as a wheel component (**39**) bearing a benzo [18]crown-6 (B18C6) macrocycle as a K^+^ selective receptor. The space length (3.43 nm) between the two ammonium stations of **37** was close to the thickness of the phospholipid bilayer, thus it enabled the proper membrane-spanning movement of rotaxane. Large unilamellar lipid vesicles (LUVs) assay and lipid bilayer membranes (BLMs) experiments were conducted to explore the ion transport activity as well as the gated behavior of **37**. The experimental results indicated that the ring could reversibly shuttle between two ammonium stations when the AB unit was in its *E*-form (corresponding to visible light irradiation), leading to the efficient K^+^ ion transport. After UV light irradiation, the corresponding ***Z*-37** exhibited much lower transmembrane activity owing to the limited movement of the macrocycle in the *Z*-isomer. Notably, this example is the first MIM used for light-gated ion transport based on a synthetic molecular shuttle.

### Molecular Muscles

By combining the molecular shuttle and doubly interlocked rotaxane dimers (i.e., molecular [c2]daisy chains), the resultant MIMs can behave with stimuli-responsive contraction and extension motions. Such systems have been coined as “molecular muscles” owing to it is similar to the sarcomeres present in muscles (Bruns and Stoddart, 2014; Antoine et al., 2019). In early reports, Dawson et al. (2008) and Li et al. (2010) had already proved that single molecular [c2]daisy chains with stilbene or AB moieties could be utilized as light-driven molecular muscles in solutions.

**FIGURE 14:**
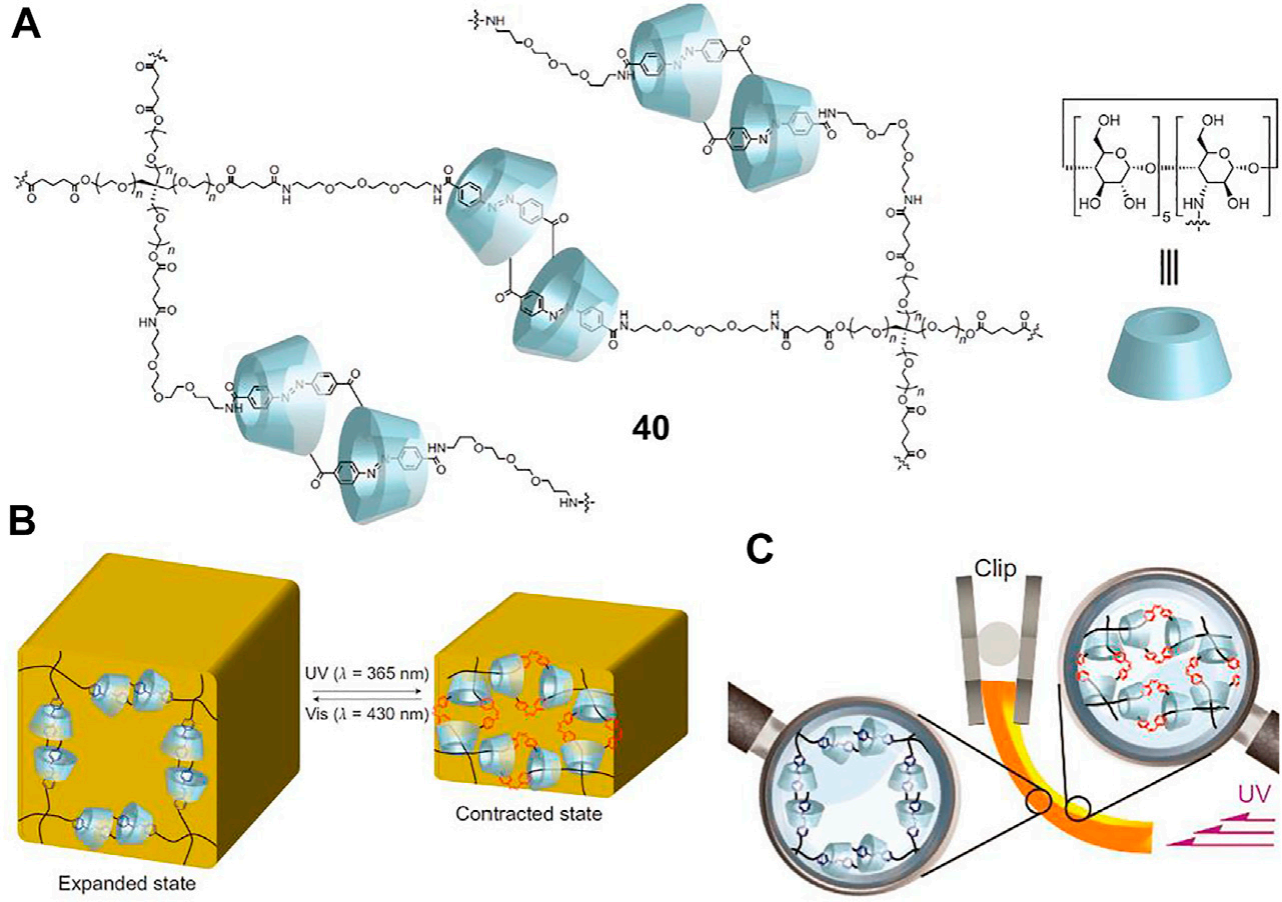
**(A)** Chemical structure of crosslinking [c2]daisy chains **40**; **(B)** schematic illustration of the light-driven volume change of **40** hydrogel; **(C)** the bending mechanism of **40** hydrogel upon photoirradiation. Adapted with permission from Iwaso et al. (2016). Copyright (2016) Springer Nature.

To overcome the challenge of macroscopic molecular actuators, Iwaso et al. (2016) designed a polymer network (Figure 14A, **40**) crosslinked with AB and α-CD based molecular [c2]daisy chains. Hydrogel and xerogel were prepared from **40**, and a photoresponsive volume change of hydrogel was first investigated. Upon UV light exposure, obvious volume reduction of the resultant polymer gels was observed, which could recover to the initial state after irradiation with visible light for 3 h (Figure 14B). When hydrogel and xerogel were carried out photoresponsive actuation experiments, both of them bended toward the UV light source, which was attributed to the sliding movement of the molecular [c2]daisy chain units triggered by the *trans-to-cis* photoisomerization of the AB unit (Figure 14C). Thanks to the higher density of photo-responsive molecular muscle units, the response time of xerogel upon UV light irradiation was very rapid (7° per second), which was more than 10,000 times faster than that of hydrogel (7° for 3 h). More importantly, the xerogel of **40** was successfully applied as a mechanical arm to lift a real substance using UV light irradiation as external stimulus to produce actual work. However, the **40** xerogel did not realize reversible switching by light irradiation.

**FIGURE 15:**
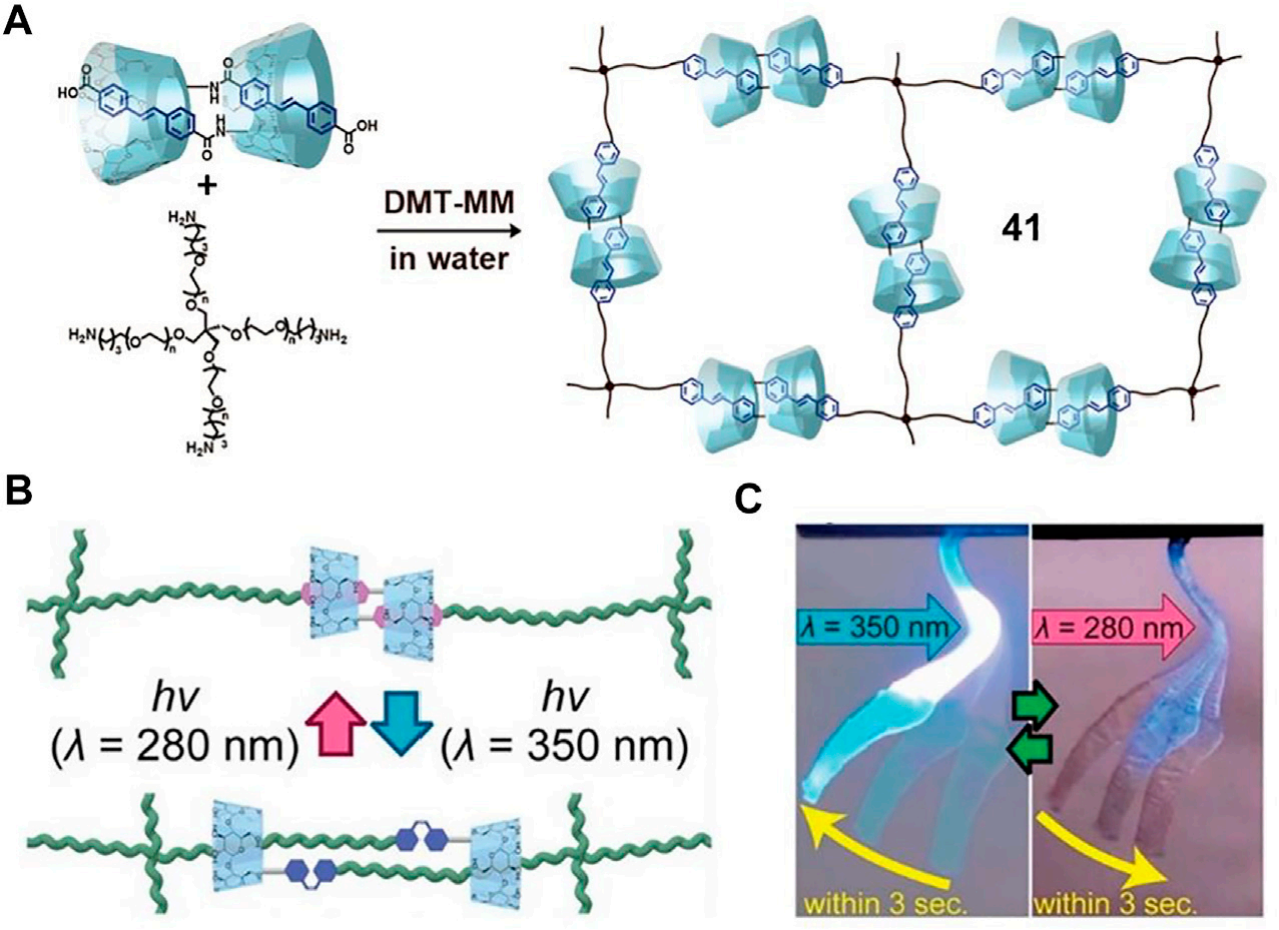
**(A)** Synthesis of cross-linking [c2]daisy chains **41**; **(B)** the photoisomerization diagram of stilbene-based **41**; **(C)** the repeatable bending of **41** xerogel by UV with different wavelengths. Adapted with permission from Ikejiri et al. (2018). Copyright (2018) American Chemical Society.

To further enhance the responsive speed and reversibility of macroscopic motions, the αCD-AB molecular [c2]daisy chain was subsequently replaced by a more sensitive cross-linking αCD-stilbene [c2]daisy chain (Figure 15A, **41**) (Ikejiri et al., 2018). The *trans*-stilbene moieties in the [c2]daisy chains could reversibly form dimeric aggregation and be dissociated by photoisomerization (Figure 15B). When subjected to 350 nm irradiation, the **41**-based dry gel (molecular weight: 20,000) rapidly bended to 26.0° within 3 s, and it returned to its original state within 3 s upon 280 nm irradiation, leading to a reversible actuation process (Figure 15C). Actually, the hydrophobic feature of the stilbene unit might improve the reversible switching ability of [c2]daisy chains. Finally, the dry gels could perform quick and heavy mechanical work upon light irradiation, which picked up an object 15 times heavier than its own weight.

## Summary and Outlook

As typical molecular machines, molecular shuttles could produce nanoscale mechanical shuttling motions of wheel components in response to external stimuli, making them privileged platforms for the construction of artificial molecular machines. In particular, light is a very attractive resource to drive molecular shuttles owing to its clean, cheap, directional, and controllable features, thus light-responsive molecular shuttles have attracted more attention. The processes of light-driven molecular shuttle motions involve several mechanisms: photoinduced isomerization, photochemical reaction, and photoinduced electron transfer. The photoinduced isomerization could be gained based on N=N double bond, C=C double bond, or C=N double bond. Several reversible photochemical reactions have been successful in constructing molecule shuttles, and one obvious feature of photochemical reactions is that they would release or require the addition of small molecular reagents to complete the whole reversible processes. Moreover, photochemical reactions can sometimes change the microenvironments surrounding rotaxanes, which could be applied to drive non-photoresponsive molecular shuttles. The photoinduced electron transfers usually exist in donor-acceptor systems and need sacrifice reagents to assist supplementary completion. Over the past decade, researchers pay more attention to realize the practical applications of light-driven molecular shuttles. The light-driven molecular shuttles have been applied in optical information storage, drug delivery, catalysts for organic synthesis, ion transport, and molecular muscles, which opens a wide door to the nanoscience.

Although great achievements have been realized in this area, the further expansion of the applications of light-driven molecular shuttles will still be in great demand. Some key issues remain to be addressed: 1) improving the conversion efficiency, residence time, and switching ratio of different states; 2) expanding the reaction types, improving catalytic efficiency and selectivity, and realizing multiple recycling of catalysts; 3) achieving a wider range of biological applications, such as improving not only the controllability and types of drug release, but also the efficiency, sensitivity, and selectivity of ion transport processes; and 4) constructing proper communication bridges between microscopic molecular shuttles and macroscopic devices, and fabricating practical devices based on light-driven molecular shuttles.
